# Influences of Vehicle Communication on Human Driving Reactions: A Simulator Study on Reaction Times and Behavior for Forensic Accident Analysis

**DOI:** 10.3390/s24144481

**Published:** 2024-07-11

**Authors:** Maximilian Bauder, Daniel Paula, Claus Pfeilschifter, Franziska Petermeier, Tibor Kubjatko, Andreas Riener, Hans-Georg Schweiger

**Affiliations:** 1 Technische Hochschule Ingolstadt, CARISSMA Institute of Electric, Connected, and Secure Mobility, Esplanade 10, 85049 Ingolstadt, Germany; daniel.paula@thi.de (D.P.); franziska.petermeier@gmx.de (F.P.); hans-georg.schweiger@thi.de (H.-G.S.); 2 Technische Hochschule Ingolstadt, CARISSMA Institute of Automated Driving, Esplanade 10, 85049 Ingolstadt, Germany; claus.pfeilschifter@thi.de (C.P.); andreas.riener@thi.de (A.R.); 3 Institute of Forensic Research and Education, University of Zilina, 010 26 Zilina, Slovakia; tkubjatko@gmail.com

**Keywords:** vehicle-2-X communication, accident analysis, accident reconstruction, human factors, reaction times, fundamental data

## Abstract

Cooperative intelligent transport systems (C-ITSs) are mass-produced and sold in Europe, promising enhanced safety and comfort. Direct vehicle communication, known as vehicle-to-everything (V2X) communication, is crucial in this context. Drivers receive warnings about potential hazards by exchanging vehicle status and environmental data with other communication-enabled vehicles. However, the impact of these warnings on drivers and their inclusion in accident reconstruction remains uncertain. Unlike sensor-based warnings, V2X warnings may not provide a visible reason for the alert, potentially affecting reaction times and behavior. In this work, a simulator study on V2X warnings was conducted with 32 participants to generate findings on reaction times and behavior for accident reconstruction in connection with these systems. Two scenarios from the Car-2-Car Communication Consortium were implemented: “Stationary Vehicle Warning—Broken-Down Vehicle” and “Dangerous Situation—Electronic Emergency Brake Lights”. Volkswagen’s warning concept was utilized, as they are the sole provider of cooperative vehicles in Europe. Results show that V2X warnings without visible reasons did not negatively impact reaction times or behavior, with average reaction times between 0.58 s (steering) and 0.69 s (braking). No significant distraction or search for warning reasons was observed. However, additional information in the warnings caused confusion and was seldom noticed by subjects. In this study, participants responded correctly and appropriately to the shown false-positive warnings. A wrong reaction triggering an accident is possible but unlikely. Overall, V2X warnings showed no negative impacts compared with sensor-based systems. This means that there are no differences in accident reconstruction regarding the source of the warning (sensors or communication). However, it is important that it is known that there was a warning, which is why the occurrence of V2X warnings should also be saved in the EDR in the future.

## 1. Introduction

Cooperative intelligent transport systems (C-ITSs) are vehicles that exchange information directly with other C-ITSs [1]. Depending on the communication partner, a distinction is made between vehicle-to-vehicle (V2V), vehicle-to-infrastructure (V2I), and vehicle-to-person (V2P) communication, which can be summarized by the general term vehicle-to-X (V2X) communication [2]. The current communication development stage, also called Day 1, aims to warn the driver of possible critical driving situations to increase vehicle safety [3]. Due to the large possible communication range of up to 800 m [4,5], a dangerous situation can be detected before it is perceived by the vehicle’s environment sensor system or the driver, and a warning can be sent. This represents a major difference from conventional warning systems based on vehicle sensors. If an accident occurs despite warnings and is investigated by an accident analyst, the local and temporal context must be reconstructed as part of the avoidability consideration. Standard reaction times of the driver are used for this purpose [6]. Therefore, this work aims to investigate the change in the drivers’ reaction behavior and the reaction times when V2X warnings occur. This work will clarify whether considering V2X warnings is necessary for future accident reconstructions. The question also arises whether such systems influence the course of the accident or can be theoretically the cause of the accident, which was already proposed in [7]. The following defines driver reaction time for better understanding, and the specific research questions are formulated.

### 1.1. Driver Reaction Time

**Figure 1:**
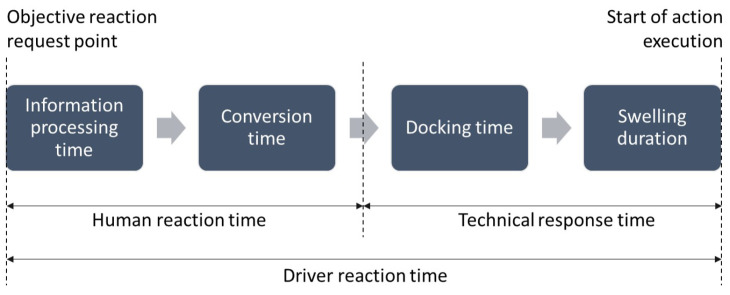
Time components of the driver reaction time according to [6,9].

Driver reaction time is defined from the beginning of the objective reaction request point until the start of a desired action execution by the vehicle [6], which can be seen in Figure 1. The driver’s reaction time can be divided into a human and a technical temporal part. The human temporal part consists of information processing time and, if applicable, conversion time. According to [8], the conversion time is understood to be the foot-to-pedal movement time for a braking or accelerator reaction or the hands-on time for the steering reaction. The hands-on time is not present when determining the steering reaction times in this study, as the test subjects drive themselves and, therefore, always have their hands on the steering wheel. The docking time corresponds to the time until the foot or hand is fully in contact with the pedal or steering wheel and the first movement of the pedal/steering wheel begins. The swelling duration then describes the time until the vehicle actually starts to convert the system input.

According to the current state of the art, braking reaction times between 0.8 s to 1.3 s and steering reaction between 0.6 s to 1.2 s, or sometimes even more, are applied by experts in accident reconstruction, depending on the situation and the kind of reaction (steering or braking) [6]. An overview of possible driver reaction times out of 160 studies is given in [10]. Since it is not possible to prove whether and when a warning was given by the vehicle to the driver and whether the driver also noticed it [11], a warning is currently not considered when setting the reaction time for reconstructing accidents. The reaction time also depends on the factors of urgency and the driver’s expectation of the occurrence of the event, which are varied in the course of the study by using the warnings [10]. However, other influencing factors such as age, gender, distraction, and environment were excluded in this study to measure the actual influence of V2X Warnings on reaction time and behavior. This was ensured in the design of the scenarios.

### 1.2. Related Work

The first studies on the influence of vehicle communication on drivers were already conducted in 2016. In [12], the lead time for the optimal timing of a V2X warning was investigated. The study took place in an urban scenario with 30 test subjects, whereby each test subject ran 16 collision scenarios. The results show that warnings 5–8 s before the collision provided the greatest improvement in road safety. Unlike our study, no reaction times and behaviors were investigated by [12]. Furthermore, the warning concept at that time did not correspond to the warning concept of real V2X Vehicles. In contrast, our work examines the actual warning concept used in Volkswagen’s warning tone and images. The environment and the associated distractions caused by other objects in the city also differ from those in our study.

In [13], the effects of false and unnecessary warnings by cooperative vehicles on drivers’ compliance were investigated. The study was conducted in an urban scenario with 80 subjects, some with a reliable warning system and some with an unreliable one. As a result, false warnings were found to negatively affect the systems’ effectiveness and drivers’ acceptance of the system. On the other hand, it was found that false alerts were better accepted when the reason for the alert was understandable but unnecessary from the driver’s point of view. In contrast to our work, it was not investigated whether false warnings could cause accidents when drivers take actions that are unpredictable to following traffic.

The dissertation by [14] investigated the effects of auditory warnings on vehicle-to-vehicle collision warning systems. The dissertation investigated auditory warnings that greatly affect drivers’ accident avoidance. Two simulator studies were conducted. The first study presented a scenario in longitudinal traffic and a possible rear-end crash. The second scenario took place at an intersection. As a result, it was found that the urgency of the acoustic warning influences the reaction times in longitudinal traffic. The stronger the auditory warning, the faster the reaction of the drivers. Many other factors play a role in the intersection scenario. Driver reaction behavior is generally described as complex and dependent on many factors. Different from our study, the systems on the market were not investigated. Furthermore, no false warnings or warnings without a visual reason for the warning were investigated. The scenarios themselves were also different.

In [15], the parameters of warning lead time, reliability of the warning, and style of the warning were investigated by V2X warnings based on collision rates to develop recommendations for the design of the warnings. A simulator study with 32 test subjects was conducted for this purpose. The result shows that notification warnings should be used for longer warning times. Command warnings, on the other hand, should be preferred for shorter warning times. However, the study did not investigate reaction times or types of reactions.

In [16], the effects of the V2X use cases Emergency Electronic Brake Lights (EEBL), Emergency Vehicle Warning (EVW), Roadworks warning (RWW), and Traffic Condition Warning (TCW) on driving behavior, usefulness, and acceptance were investigated.

A driving simulator study was conducted with 36 subjects who experienced warnings with different urgency in critical motorway situations. The EEBL scenario is particularly interesting because it is similar to the second scenario in our study. It was also investigated whether there is a behavioral difference when the driver sees and does not see the braking vehicle. This aims to address a similar research question to the first scenario in our study. As a result of the EEBL scenario relevant to our study, it was found that the reaction times to apply the brake became smaller due to the warning. In addition, the mean speed decreased during the scenario. Both effects could be observed with and without visual feedback from the braking vehicle. Contrary to our study, systems, and warning concepts on the market were not investigated. In addition, no false warning was investigated in the EEBL scenario. Also, the lack of visual feedback was performed on a different use case in our study.

In the latest study from [17], the influence of different warning times on the situational awareness of subjects with V2X warning systems was investigated. For this purpose, the situational awareness of 40 test persons was investigated in a simulator study with noncritical scenarios at different warning times. The results show that larger warning times reduce situational awareness during normal driving events without warning, as drivers rely on the warning. The study thus shows that despite improving road safety, warnings must be used sparingly in critical situations. Otherwise, drivers’ situational awareness will deteriorate in normal situations.

### 1.3. Research Questions

Based on the related work, it is evident that some research has already been conducted concerning human–machine interaction and response behavior with the V2X system. However, the influence of the lack of visual feedback for the vehicle driver on a warning by a V2X system has not yet been investigated in detail in the field of accident analysis, leading to the first research question:

*How do vehicle drivers react to a warning when the warning reason is not visible, and how does this impact the driver’s reaction time?*

Thus, the first research question investigates the direct effects of a lack of visual feedback from the warning reason. The thesis is that the warning can confuse the driver due to the missing visual feedback and that the driver searches for a warning reason. This can be investigated using the reaction behavior. If the warning leads to confusion and distraction from the driving task, this can negatively affect reaction times and is also investigated in the course of the first research question. To clearly determine the influence of the warning in the first scenario, only half of the test subjects received a V2X warning.

In the further course of the test drive, all subjects experience a false-positive warning. Based on the first scenario, the following research question will be investigated:

*How do vehicle drivers react to a false-positive warning when visual feedback is present?*

After half of the subjects had gained a first experience with the system and built trust in it, it was investigated, which source of information (V2X warning or visual real information) was trusted more. The scenario used to answer the research question is generally intended to determine whether subjects respond to a false-positive warning. In addition, whether the previous experience leads to a different reaction behavior to the warning in half of the subjects will be investigated. The hypothesis is that the previously warned group has already built up trust in the V2X warnings and is more likely to respond to them. From this, statements can be made about the theoretical technical accident causality of the systems.

The investigation of these research questions using a real V2X warning concept has not been conducted before in the literature and represents a novelty in the field of accident analysis.

## 2. Methods and Materials

### 2.1. Participants

A total of 32 subjects with an average age of 29.3 years (standard deviation (σ) = 4.98 years) in a range between 24 and 46 participated in the study. Previous studies [18,19] show that reaction time increases in older people. According to [18], there is a correlation of an age-related increase in reaction time from age 55 on. Therefore, no elderly subjects were selected for the study to eliminate the age factor. All subjects had a valid Class B vehicle license and an average driving experience of 11.5 years (σ = 4.99 years). Just under half of the subjects reported an annual driving mileage of over 10,000 km. For a gender-independent assessment of the results, half of the subjects were male (16) or female (16). No knowledge regarding V2X communication was assumed in the selection of subjects. The study took approximately 60 min to complete for each subject. Because another study [8] was conducted simultaneously with the same subjects alongside this study, the test blocks were conducted alternately with randomly assigned subjects to avoid systematic bias due to the study procedure (Latin square design). This study was reviewed and approved by the Ethics Committee (Institutional Review Board) of the University of Zilina in Slovakia before implementation.

### 2.2. Driving Simulator

**Figure 2:**
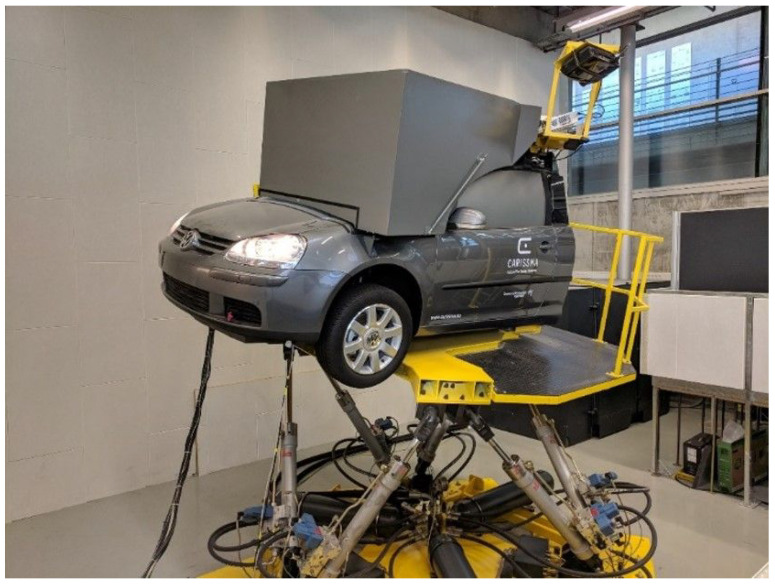
Driving simulator on a hexapod test rig at Technische Hochschule Ingolstadt [20].

The experiment was realized in a high-fidelity driving simulator simulator at Technische Hochschule Ingolstadt (see Figure 2). The simulator consists of a truncated Golf 5 placed on a hexapod with all six degrees of freedom. A highly immersive simulation environment is created with the help of 4 projectors mounted around the simulator (at the side and behind). Two projectors project the views to the front and diagonally to the side inside the box, which is mounted in front of the windscreen. The two additional side projectors project the side views, including side mirrors, directly onto the foiled window of the Golf 5. A screen on the inside wall of the cut-off rear shows the view from the rear window. [20]. The technical data of the driving simulator can be found in Table 1. The simulator’s functionality has already been proven in many studies in recent years [21,22,23].

**Figure 3:**
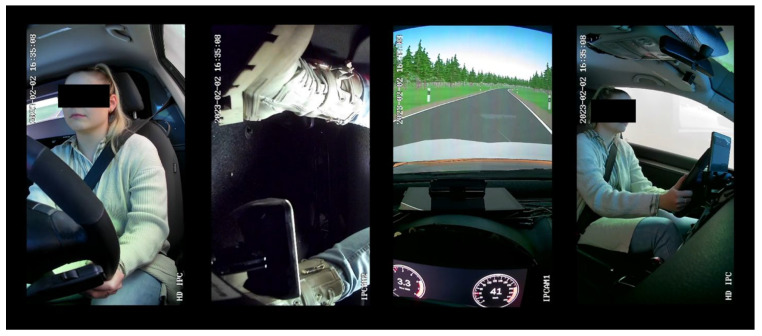
Camera views while conducting the study, from left to right: view of driver from the front, view of feet and pedals, view of the speedometer and windshield, and side view of the subject.

**Figure 4:**
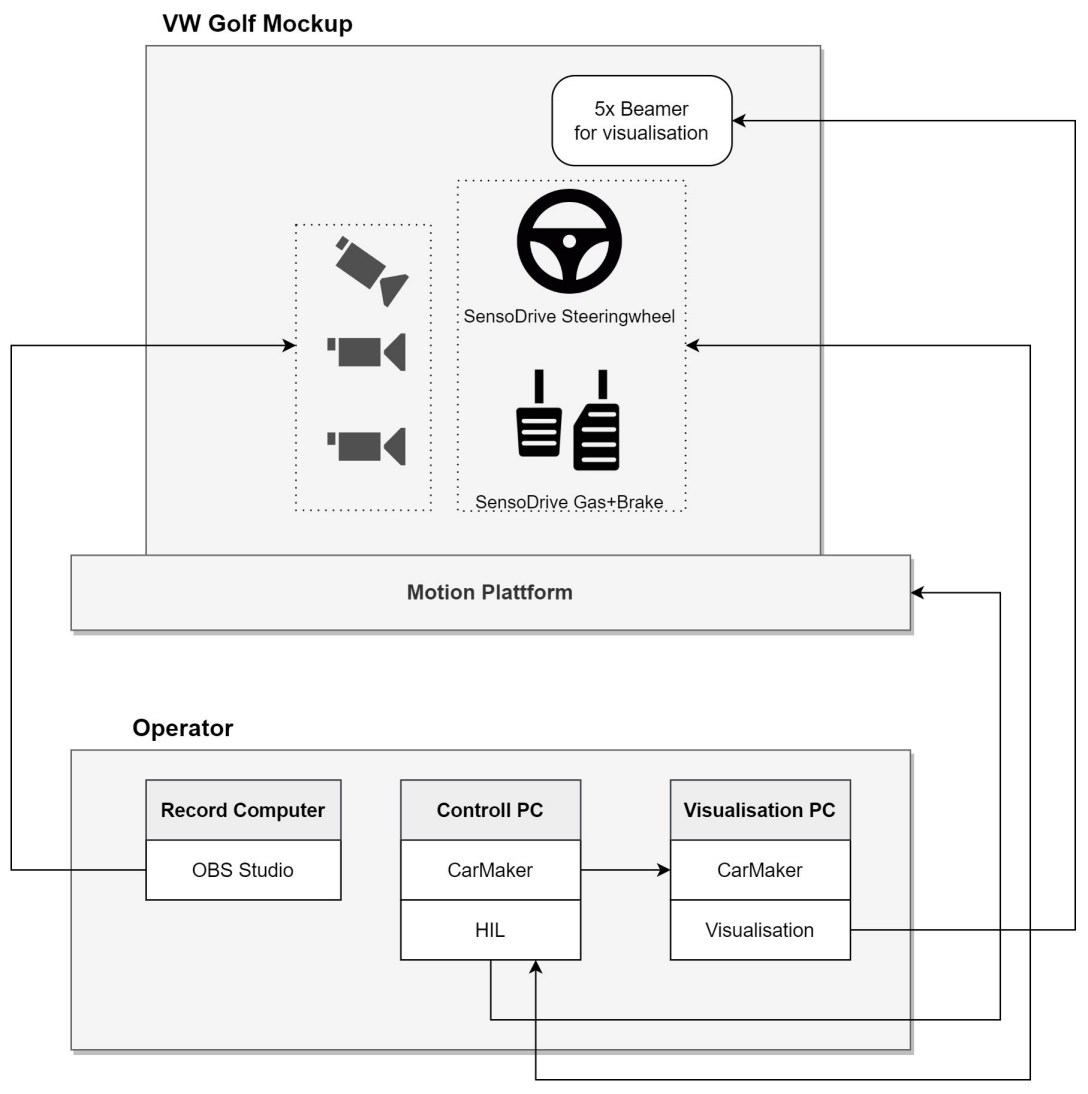
Diagram of the software/hardware setup.

IPG CarMaker 10.2.2 was used as simulation software [24]. The routes and maneuvers were completely created using IPG CarMaker. The data exchange from the simulation software to the instrument cluster was solved by using Message Queuing Telemetry Transport (MQTT) to realize the display of the warnings almost in real time. We used the driven distance to trigger the display of the warnings in each scenario. The inside was recorded with the help of 4 cameras, whose field of view can be seen in Figure 3. A summary of the entire setup is presented in Figure 4.

### 2.3. Test Scenarios and Experimental Design

The test scenarios were designed to assume a best-case situation, and other factors influencing the reaction time and behavior were avoided. During the simulation, the test persons drove on a rural road through a forest, corresponding to a real route between Kösching and Bettbrunn north of Ingolstadt (Köschingerstraße and Forststraße). The road topology was imported directly from Google Maps into CarMaker when the scenarios were created. The permitted driving speed over the entire distance is 100 km/h. The simulation environment was designed in clear midday weather with occasional oncoming traffic. This design guarantees no distraction from other objects in the vicinity.

**Figure 5:**
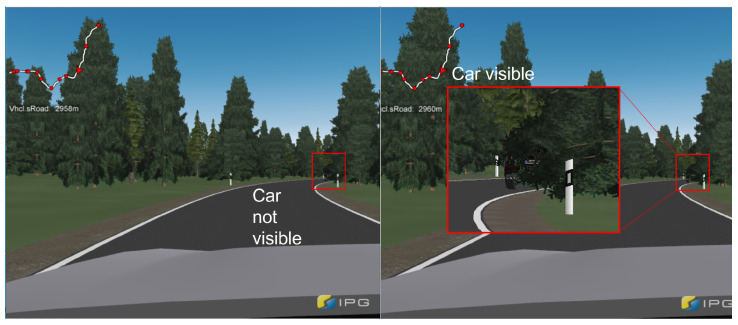
View of the driver at the reaction request point during the first scenario.

Two use cases of the Car-2-Car Communication Consortium from the Basic System Profile [25] were selected as test scenarios since they are most likely to be implemented by vehicle manufacturers and represent real situations for the scenarios. The use case “Stationary Vehicle Warning—Broken-Down Vehicle” was selected to answer the first research question, since this allowed the creation of a scenario without visual feedback under constantly the same conditions. The broken-down vehicle was placed on the sideline behind the curve and hidden by a tree. This made the broken-down vehicle visible only 44 m beforehand, as shown in Figure 5 on the right. To further increase the danger and urgency, the road was designed without a central strip, in accordance with design class 4 of the guideline for the construction of rural roads in Germany [26].

**Figure 6:**
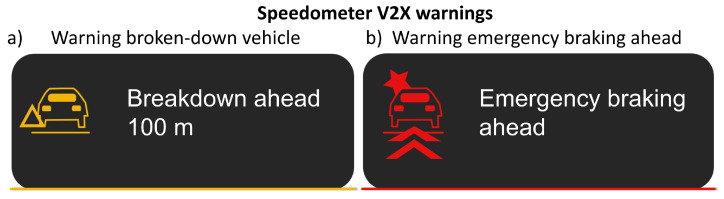
(**a**) Design of warning 100 m before the broken-down vehicle from scenario 1; (**b**) design of the false-positive warning for the electronic emergency brake light use case in scenario 2. Both warnings are according to the design of Volkswagen [5]. The warning text was displayed in German during the study ((**a**) Panne voraus 100 m) and ((**b**) Gefahrenbremsung voraus).

To investigate the influence of the V2X warning on driving behavior and reaction times, half of the subjects received an initial visual and one-time audible warning on the speedometer 250 m before the broken-down vehicle, representing the study’s independent variable. The warning here matches the design and tone of Volkswagen [5] to test a real-world implementation and is exemplified by the 100 m warning in Figure 6a. The warning was displayed continuously from this point until the distance dropped below 0 m. Every 50 m, the displayed distance to the broken-down vehicle was also updated, but without a new audible warning.

**Figure 7:**
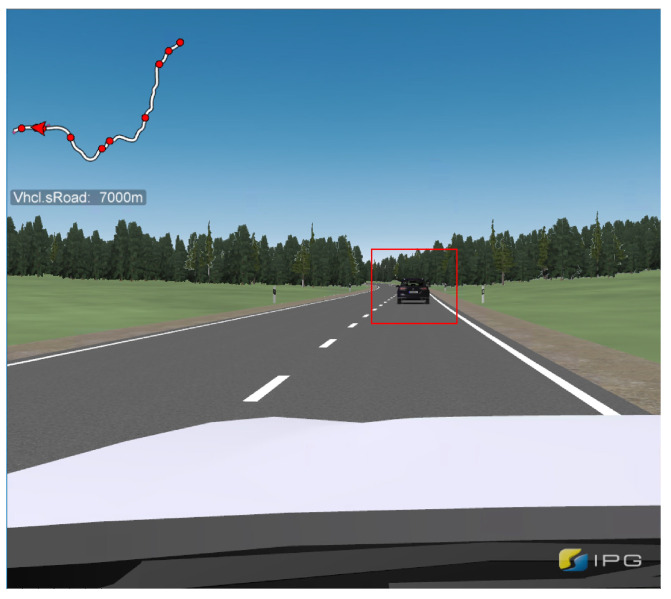
View of the driver at the reaction request point during the second scenario.

The use case “Dangerous Situations—Electronic Emergency Brake Lights” was selected to answer the second research question. In this case, the test person drives up to a slower vehicle in front and must follow it without being able to overtake. As the participants drove themselves, the distance between the vehicles could not be defined. At a certain driving distance, all subjects experienced a false-positive warning, according to Figure 6b, which implies hazardous braking of the front car. This is intended to create a situation with a high stress level and urgency due to a possible emergency braking of the vehicle in front. This should provide information about whether such a situation leads to a braking of the test persons, which could theoretically result in an accident. An example representation of the situation from the subject’s view of the vehicle ahead at the time of the warning can be seen in Figure 7. The two scenarios are always driven in the same order by all 32 subjects. This makes it possible to determine whether there is a difference between the groups with and without scenario 1 warnings. The experience with the V2X warnings from scenario 1 and the initial trust that the already warned group may have established is the independent variable for scenario 2.

### 2.4. Procedure and Instructions

Before the study, all subjects received a service agreement and an Information, Consent, and Data Privacy Sheet to read through and sign. Upon arrival at the laboratory, all subjects received a safety briefing on the laboratory and the simulator and were informed that they could stop the simulation at any time.

Subsequently, all subjects received an information sheet on how V2X communication works, analogous to the description in the Volkswagen manual [27]. Then, the subjects took their seats in the simulator and adjusted their seat according to the desired driving position. Before the simulation began, gaze instructions were given to the subject and filmed to provide a reference for later eye tracking. After that, the simulation was started and the test subjects completed any number of introductory laps to familiarize themselves with the driving simulator. Once the subject felt confident, the experimental ride started with the two scenarios, with and without the V2X warning in the first scenario. No further instructions were given to the drivers during the drive. After the simulation of both scenarios, the subjects were asked to complete a questionnaire about their experience. The experimental ride took approximately 15 min for each subject.

### 2.5. Questionnaire

With the help of Google Forms, a questionnaire was created for the test subjects, which they completed directly on site after leaving the simulator. The first part of the questionnaire contained questions about the first scenario and differentiated between the warned and unwarned groups. The unwarned group was first asked whether they were surprised by the situation, which they could answer with “yes” or “no”. They were then given the opportunity to write a reason in a free text field. Next, the test subjects were asked whether they would have found a warning helpful, again with a yes/no question and subsequent opportunity to give reasons. Finally, they were asked to rate the criticality of the scenario on a scale from 1 (very uncritical) to 5 (very critical). The warned group was also asked questions about the V2X warnings shown in scenario 1. They had to answer yes or no as to whether the warnings in general and the additional information they contained were helpful, which they could then explain in a free text field. Finally, they were asked whether they had noticed the change in the additional information (yes/no).

The second part of the questionnaire for scenario two, on the other hand, was the same for all test subjects. Here, the subjects were first asked whether they reacted to the situation in their opinion (yes/no) and why. Then the subjects were asked to rate the criticality of the situation (from 1 very uncritical to 5 very critical) and the placement of the warning (from 1 very good to 5 poor) and to give a reason for each. The test subjects were then asked whether they would switch off the system if such situations would occur more frequently. Finally, they were asked to state (yes/no) whether such situations should be practiced in driving school training.

### 2.6. Dependent Variables

During the study, the measured variables presented in Table 2 were recorded. To answer research question 1, part 1, *How do subjects react to a warning when the reason for the warning is not visible?,* the camera data on eye tracking in particular were examined. Here, an analysis of variance (ANOVA) was performed between the two study groups in the time interval from the first appearance of the warning to the appearance of the broken-down vehicle. Specifically, the proportions of time and the gaze frequency that the subjects spent looking at the simulator speedometer, the lateral environment (looking to the right on the screen), the road guidance (looking straight ahead on the screen), and other fixed points (rearview mirror, side mirror) are accumulated. This allows one to deduce whether the warning leads to increased distraction. At the same time, the brake and accelerator pedal positions and driving speed between the groups are analyzed to conclude a more careful and anticipatory driving style of the test persons due to the warning. The warning’s usefulness, presentation, and comprehensibility were also investigated using the questionnaire after the study.

To answer research question 1, part 2, *What is the effect of the warning on the driver’s reaction time?,* the brake and accelerator pedal positions, as well as the parameters of the steering wheel (angle, speed, and acceleration), are analyzed using ANOVA between groups. Here, reaction time is defined as the time interval from the appearance of the broken-down vehicle in the curve (objective reaction response point) to a defensive response of the driver. The objective reaction request point was always at the same location (distance traveled) for all test subjects in each scenario. The braking reaction times were measured from the moment the brake pedal position was no longer zero or changed by more than 1%. For the steering reaction time, the point in time was selected when the previously set steering angle changed by 0.01 rad (≈0.6°) and the steering behavior observed in the video can be recognized from the further sequence of the data. The recorded data from CarMaker with the video data was validated for all test subjects for each reaction.

The reaction behaviors distinguished in this study are shown in Table 3. The determined reaction times are also compared with those in the literature. Furthermore, the type of defensive reaction between the two groups is analyzed.

To answer research question 2, *How do vehicle drivers react to a false-positive warning when visual feedback is present?*, the eye tracking data, pedal positions, and steering wheel activities are again analyzed. As with research question 1, part 1, temporal gaze proportions are analyzed, and the reaction behavior is again evaluated according to Table 3. Since half of the group had experience with the system, while the other group was new to V2X warnings, it was investigated if the experience from scenario 1 led to different results in scenario 2. The analysis was also verified by applying an ANOVA. In addition, a questionnaire was used to collect information about the criticality feelings of the subjects in this situation to explain possible reactions and nonreactions better.

The postsimulation questionnaire consists of 20 questions for the subjects with warning and 16 questions for the subjects without warning in the 1st scenario. Yes/No and rating questions were asked with a range of 1 to 5, followed by free text answer options for each question.

## 3. Results and Discussion

### 3.1. Scenario 1: Broken-Down Vehicle Warning

**Figure 8:**
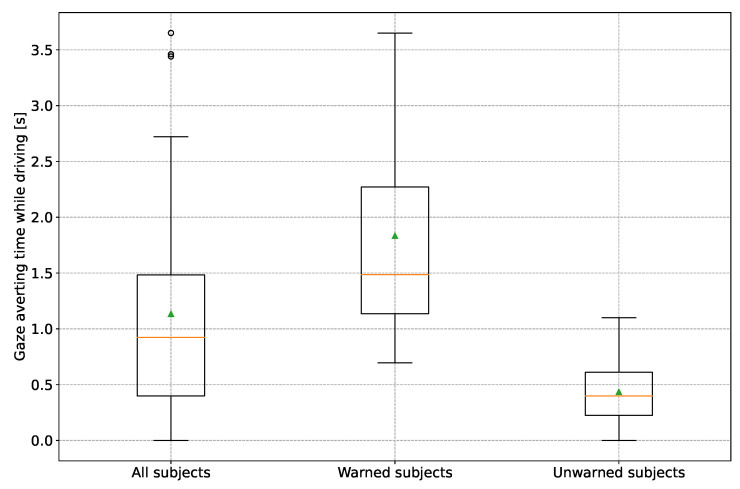
Boxplot diagram of cumulative time proportions of gaze averted from the road from the start of the warning to the appearance of the broken-down vehicle. The orange line indicates the median and the green triangle the mean.

To answer research question 1, part 1, *How do subjects react to a warning when the reason for the warning is not visible?*, the time proportions of gaze averts from the beginning of the first warning to the appearance of the broken-down vehicle were cumulated. Figure 8 shows the result of this evaluation as a boxplot diagram. The boxplot for all test subjects is shown on the left. In the center is the boxplot for the warned test subjects and on the right is the boxplot for the unwarned test subjects. The same applies to all subsequent boxplot diagrams in this work.

It can be seen that on average (median = 1.49 s/mean = 1.83 s), the warned group averts its gaze from the driving task for a significantly longer time than the unwarned group (median = 0.40 s/mean = 0.43 s). Using an ANOVA, it could be statistically validly demonstrated with a *p*-value of $8.05\times 10^{-6}$ at a significance level of α=0.05 that the warned group took significantly longer to avert their gaze from the driving task. No gender difference could be found when the Tuckey test (for definition see [28]) was applied.

Further analysis showed that the subjects of the warned group almost exclusively looked at the speedometer, which displayed the warning, as a reason for averting their gaze. Only two subjects looked at the surroundings and the rearview mirror. Thus, it can be generally stated that the warning leads to an increased gaze aversion from the driving task compared with the unwarned group. One hypothesis was that the subjects might be confused about the warning reason due to the lack of visual feedback and search for the warning in the environment, if necessary. This could not be determined. The effects of the increased gaze averting time from the driving task but also increased attention due to the warning on the reaction time and behavior to the broken-down driver are shown in the following.

**Figure 9:**
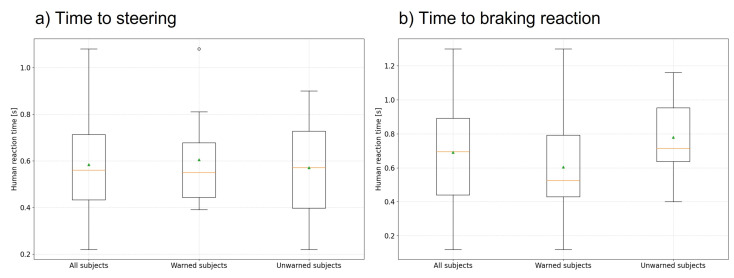
Boxplot diagram of reaction times, the orange line indicating the median and the green triangle the mean: (**a**) Distribution of measured steering reaction times. (**b**) Distribution of measured braking reaction times.

Figure 9 shows the result of the measured braking and steering reaction times as a boxplot diagram analogous to the distribution in Figure 8. The left diagram shows the steering reaction times, and the right diagram shows the braking reaction times resulting from the different reaction types.

A distinction is necessary because, with one exception, the unwarned group, in particular, reacted with a steering and braking maneuver (see Figure 12), and the reaction times differ between these reactions. Thus, in most cases, the steering reaction occurred faster than the braking reaction due to the additional conversion time between the brake and gas pedal pedals. This has also been noted previously in [10]. For the warned group, only a small distinction in reaction times could be determined for a combined reaction since they were already ready to brake or were already braking due to the warning.

Thus, the mean steering reaction time across all subjects is 0.58 s, with a standard deviation σ of 0.20 s, and the mean braking reaction across all subjects is 0.69 s, with a standard deviation σ of 0.28 s. The warned group’s mean values are 0.61 s (σ = 0.22 s) for the steering reaction and 0.60s (σ = 0.31 s) for the braking reaction. Analogously, mean values of 0.57 s (σ = 0.20 s) for the steering response and 0.78 s (σ = 0.23 s) for the braking response are obtained for the unwarned group.

The hypothesis, and thus the second part of research question 1, that reaction times increase due to the lack of a warning reason and increased distraction due to the warning, was again examined using an ANOVA at a significance level of α=0.05. The result of the ANOVA shows no significant difference in the reaction times of the two groups (*p*-value = 0.68 for steering reaction times and *p*-value = 0.10 for braking reaction times). There is also no difference between the genders here either.

The fact that, as in the case of [16], the reaction times are shorter due to the warning compared with the unwarned group could, therefore, not be confirmed here. This could be due to the scenario’s design as a best-case scenario since no lowered attention or distraction can be assumed for the unwarned drivers. Thus, the effect of the warning on reaction times is small.

Regarding the lower measured reaction times than usual in the literature, the best-case scenario can also be named as a reason. In addition, there were small inaccuracies in evaluating the reaction times by the measuring equipment. For example, the temporal resolution was limited to 10 ms, so a maximum error of 5 ms can result. In addition, the objective reaction request point depends slightly on the lateral offset of the subjects on the road during the simulation. Subjects who drove a little further out on the curve could detect the broken-down vehicle about 2 m earlier, leading to an average inaccuracy in the evaluation of up to 130 ms.

**Figure 10:**
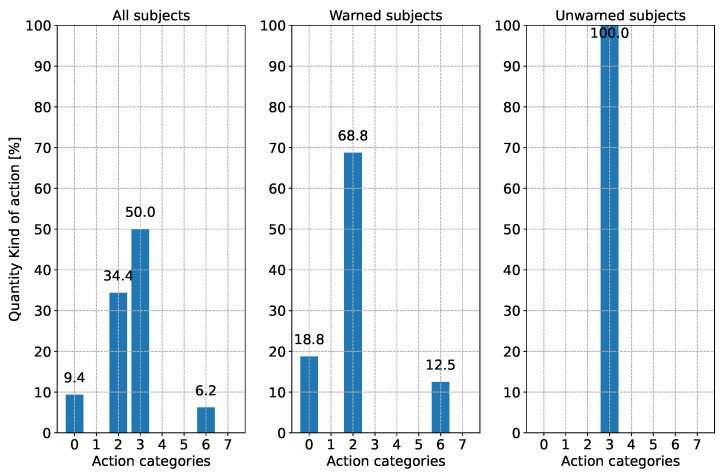
Reaction behavior according to Table 3 of the subjects when the V2X warning first appeared (250 m before the broken-down vehicle). For the unwarned group, the current driving action is shown for comparison purposes. Please note: Due to rounding errors, deviations of 0.1% from 100% may occur in total.

**Figure 11:**
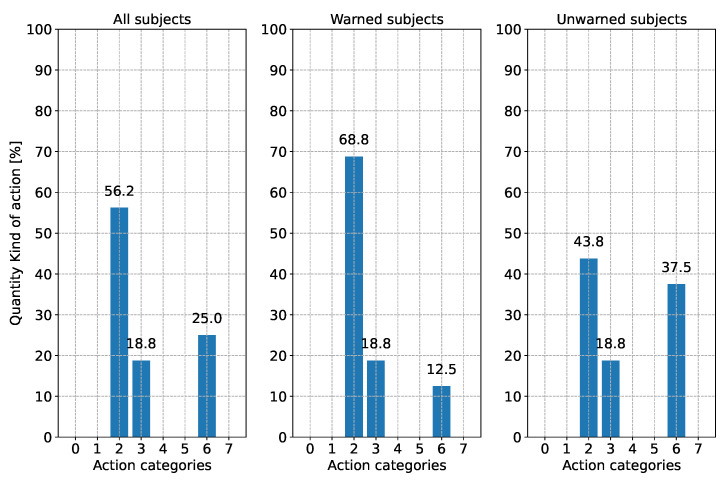
Reaction behavior according to Table 3 of the subjects shortly before the appearance of the broken-down vehicle. Due to the cornering, the driving action “steering” is not considered for both groups. Please note: Due to rounding errors, deviations of 0.1% from 100% may occur in total.

**Figure 12:**
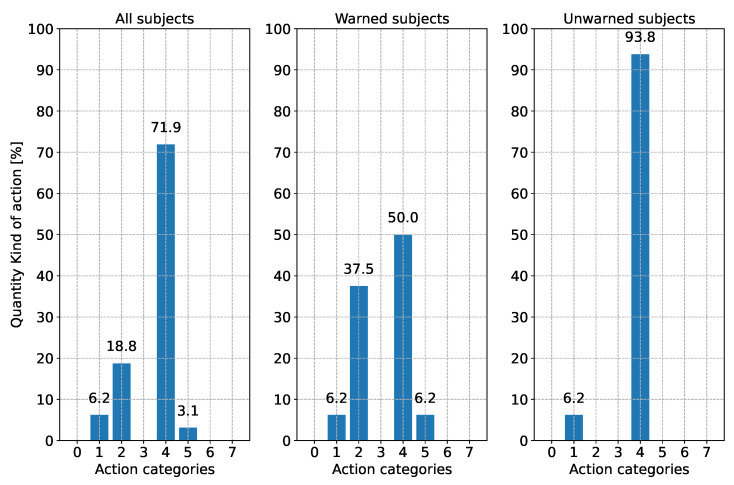
Reaction behavior according to Table 3 of the subjects when the broken-down vehicle was detected (objective reaction request point). Please note: Due to rounding errors, deviations of 0.1% from 100% may occur in total.

When evaluating the reaction type, a distinction is made between three different points in time. Figure 10 shows the subjects’ driving reaction when the first V2X warning appeared. Since this could only be evaluated for half of the subjects, the current action during the performance of the driving task by the subjects without warning at this time point is shown. In Figure 11, the subjects’ driving action is shown shortly before the appearance of the broken-down vehicle. Since the subjects drive around a curve, the reaction “steering” is excluded from this analysis. Figure 12 illustrates the type of reaction when the broken-down vehicle appears.

According to Figure 10, it can be seen that all the unwarned subjects applied the gas pedal at the time of the first V2X warning and continued to do so. The response of the warned group to the warning is that almost 70% of the subjects applied the brake, while 12.5% reduced the accelerator pedal position. On the other hand, one-fifth did not react to the warning.

Shortly before the broken-down vehicle’s appearance, the subjects’ reactions equalize (see Figure 11). Over 80% of the subjects in the warned and unwarned groups brake or reduce the accelerator pedal position (categories 2 and 6). In both groups, 1/5 of the test subjects were still on the gas. However, the background to this is completely different between the two groups. While the unwarned group was still on the throttle, as they saw no reason to brake because of the curve, the test subjects in the warned group were back on the throttle after already braking hard to avoid becoming too slow.

The resulting difference can be seen in the reactions to the broken-down vehicle in Figure 12. While in the unwarned group, all subjects had to take evasive action by steering and braking to avoid a collision due to the higher speed, more than one-third of the subjects in the warned group could avoid the collision by further braking. Half of the warned group also reacted to the broken-down vehicle by braking and steering. Also recognizable in Figure 12, there were three subjects (two subjects in the warned group and one subject in the unwarned group) who showed deviating behavior compared with the other subjects when the broken-down vehicle appeared. Despite the warning, one subject was only able to take evasive action with a steering reaction (category 1), as it was still traveling too fast (88 km/h) when recognizing the obstacle. The other warned subject, who reacted with a gas pedal and steering reaction (category 5), had already significantly reduced his speed before and had already pressed the accelerator pedal again when driving through the curve, as well as when the broken-down vehicle appeared. The other unwarned test subject, who only showed a steering reaction (category 1), was also traveling very fast (93 km/h), which led to an overload situation. This could be recognized because the subject shifted their foot back and forth between the accelerator and brake but could not decide which pedal to press. Instead, only an active steering reaction was performed. However, these reactions are isolated exceptions to the reactions of most other test subjects and do not represent the expected reaction pattern in this situation. It is not to be expected that 6.2% of the population would react in this way. The value should be treated with caution due to the sample size of 32. The percentage of people who would actually react in this way must be determined in studies with more subjects.

**Figure 13:**
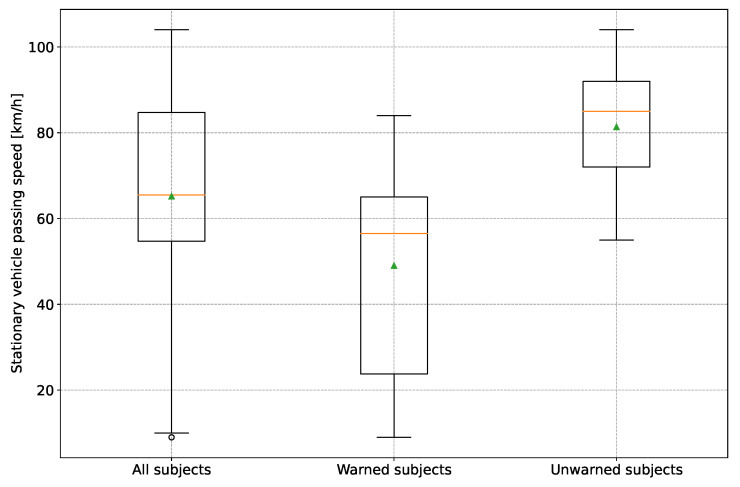
Driving speed of the test persons when passing the broken-down vehicle. The orange line indicates the median, and the green triangle the mean.

The statistical distribution of the velocities when passing the broken-down vehicle is shown as a boxplot diagram in Figure 13.

A significantly lower mean speed of the warned group (median = 56.5 km/h/ mean = 49.0 km/h) than the unwarned group (median = 85.0 km/h/mean = 81.4km/h) can be seen. ANOVA also confirmed statistical significance (*p* = 8.38 $\times 10^{-5}$). There was no discernible difference in gender. Thus, the result confirms the finding from [16] that the warning reduces the average driving speed.

**Figure 14:**
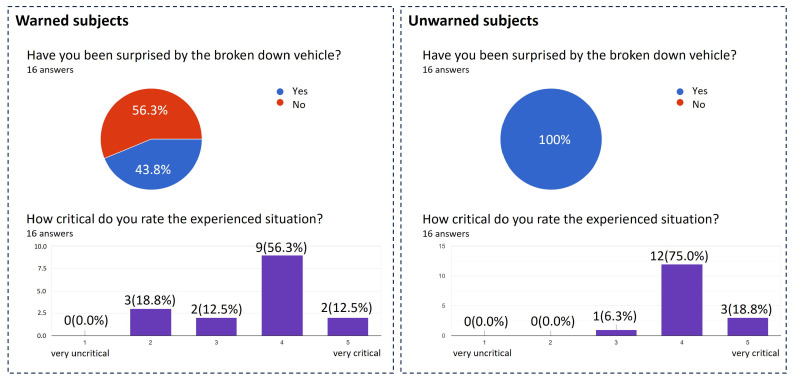
Subjects’ answers in the questionnaire on the first scenario after the study was conducted.

Finally, Figure 14 shows the answer’s evaluation of two questions from the questionnaire. A large difference can be seen in the test persons’ evaluation of the element of surprise regarding the broken-down vehicle. While all subjects in the unwarned group were surprised by the broken-down vehicle, only 43.8% of the subjects in the warned group were. Despite the warning, many warned subjects were still surprised by the broken-down vehicle. According to their explanations, this was due to the difficulty in accurately interpreting the distance indicated in the warning and the lack of visual feedback. As a result, the warning was not fully understood by some subjects. Based on these statements, it can be seen that visual feedback plays a role in estimating the warning in the driving task. However, negative influences on the reaction behavior and reaction times could not be found. In comparison with warnings of sensor-based systems, no difference is to be expected due to the lack of visual feedback with V2X warnings.

The criticality of the situation was assessed slightly lower by the subjects with a warning, which can also be explained by the lower average speed at the occurrence of the situation.

The unwarned group was also asked if they would have found a warning about the situation helpful. A total of 93.8% of the subjects agreed with such a statement. The subjects with a warning were asked in the same course whether they had found the warning helpful. All subjects agreed on that. The additional information on the distance to the critical point in the warning was found helpful by 87.5% of the subjects. The decrease in agreement with this question can be explained by the difficulty in interpreting the distance information, which some subjects included as an explanation. Two-thirds of the subjects also did not notice the changes in the additional information, which generally questions its effectiveness. However, this could also be due to the subjects’ lack of experience with V2X warnings, as these are not yet very common in vehicles on the market.

### 3.2. Scenario 2: False-Positive Warning

**Figure 15:**
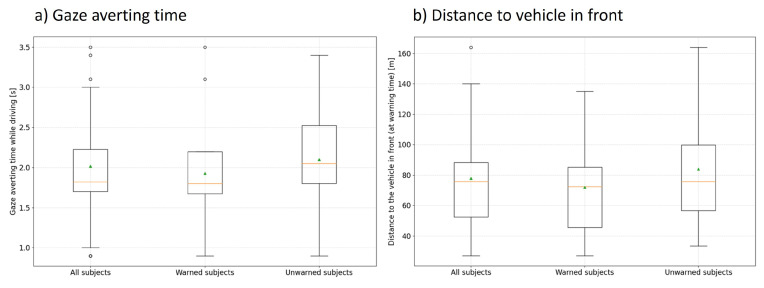
(**a**) Cumulative time proportions of gaze averts from the road at the appearance of the false-positive V2X warning; (**b**) distance of the subjects to the vehicle in front when the false-positive V2X warning appeared. The orange line indicates the median and the green triangle the mean.

**Figure 16:**
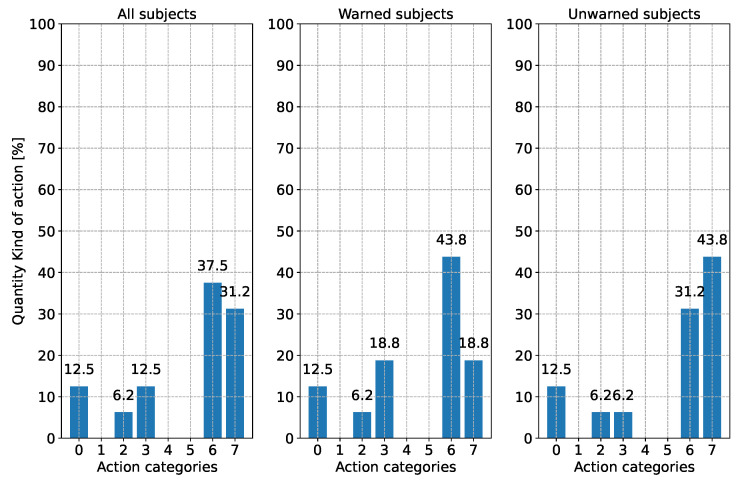
Reaction behavior according to Table 3 of the subjects when the false-positive V2X warning appeared. Please note: Due to rounding errors, deviations of 0.1% from 100% may occur in total.

To answer the second research question in scenario 2, the cumulative gaze aversion from the driving task (Figure 15a), as well as the reaction behavior to the warning (Figure 16), is evaluated again. On average, both groups were distracted from the driving task for approximately the same time. Moreover, both groups only had gaze averts to the speedometer and did not look for any other reason for the warning.

No obvious difference can be found between the subject groups in the type of reaction according to Figure 16 either. In both cases, more than two-thirds of the subjects stepped off the gas. Most of the unwarned group in scenario 1 observed the situation ready to brake. Here, the previously warned subjects let off the gas slightly less cautiously. Just under 20% of the subjects warned in scenario 1 stayed on the gas. However, it cannot be determined with certainty whether this is due to the subjects’ experience. Two subjects stated that they had reacted more cautiously because of the warning in the first scenario.

From the point of view of accident analysis, it is also interesting to see whether a false-positive warning can lead to a real reaction of the drivers. In both groups, only 6.20% of the subjects initiate a braking action due to the warning. Thus, although causing an accident is theoretically within the realm of possibility, it is rather unlikely. It should also be noted that a braking reaction does not have to lead directly to an accident, but the braking maneuver must be sudden and strong. It also depends on the distance of the vehicle and the reaction time of the driver behind. The hypothesis that more experienced subjects are more likely to believe a false-positive warning and initiate a braking maneuver cannot be proven based on the reaction behavior of the subject groups.

The reaction behavior could also be dependent on the safety distance to the vehicle in front. Again, no significant difference can be observed based on the boxplot diagram in Figure 15b. This could be confirmed by ANOVA (*p*-value = 0.33). Thus, the mean distance of the two subject groups to the vehicle in front is comparable and has no influence on the result of the reaction.

**Figure 17:**
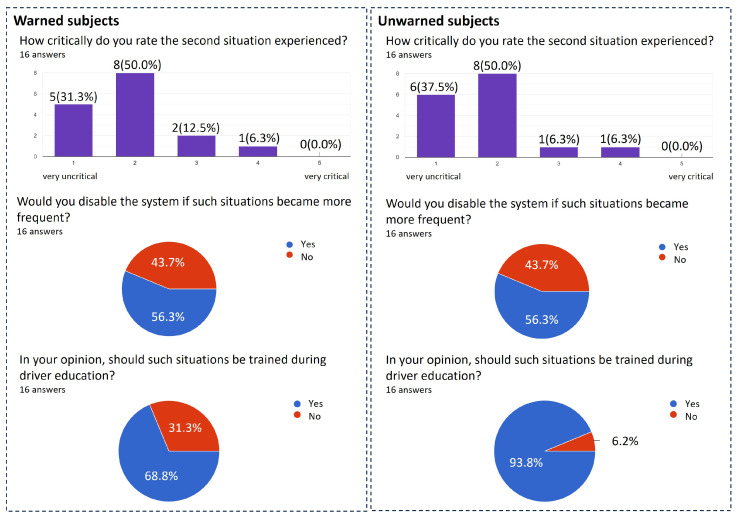
Subjects’ answers in the questionnaire on the second scenario after the study was conducted.

Finally, the answers to the questions from the questionnaire for this scenario are presented in Figure 17. When asked about the criticality of the situation, it is apparent that there is an almost identical result across both groups that the situation was rather uncritical. Uncertainty of the drivers due to the warning leading to an immediate reaction can not be determined.

Since several studies have already examined the acceptance of driver assistance systems with regard to fals-positive triggers [13,29], it was also asked whether the subjects would deactivate the system in the event of an increasing number of false-positive warnings. For both groups, just over half said they would disable the systems if they received frequent false-positive warnings.

Lastly, it was asked whether the situation of a false-positive warning should be trained in driving school. The vast majority of subjects agreed with this. From the results of this study, it can be deduced that most subjects already react appropriately to a false-positive warning. However, practice in driving school could improve the result even further.

## 4. Conclusions

In this work, the effects of V2X warnings on the reaction times and behavior of vehicle drivers were investigated, which will be used as basic data for accident analysis. For this purpose, a study with 32 subjects was conducted on the driving simulator of Technische Hochschule Ingolstadt.

Two research questions were formulated and investigated with one scenario each. Best-case parameters were chosen for the study (no distraction, no secondary activity, hardly any traffic, rural environment) to avoid further influences on the reaction times and behavior. In the first scenario, subjects had to react to a broken-down vehicle in a curve, with half of the subjects receiving a V2X warning 250 m before the broken-down vehicle. In the second scenario, a false-positive V2X warning indicating an emergency braking of a slower vehicle in front was investigated. Both warnings were based on the real V2X warning concept of Volkswagen.

The first research question, which was investigated with the scenario of the broken-down vehicle, can be divided into two parts. The first part of the question is *How do subjects react to a warning when the warning reason is not visible?* As a result of this question, it was found that the absence of the warning reason does not influence the effectiveness of the warning itself. Most subjects complied with the warning and reduced their driving speed by reducing the accelerator pedal position or braking. Increased distraction by the warning due to gaze aversion from the driving task was demonstrated but without negative effects on reaction times.

This addresses the second part of the research question, *What is the effect of the warning on the driver’s reaction time?* Regarding reaction times, mean reaction times between 0.58 s (steering reaction) and 0.69 s (braking reaction) were found. A significant difference between the warned and unwarned groups could not be determined. Thus, it can be concluded from the first scenario that the absence of a warning reason does not lead to increased driver distraction, disregard of the warning, or even an increase in reaction time due to distraction and lack of understanding. Furthermore, it was found that the element of surprise caused by the broken-down vehicle was significantly reduced by the warning. The warning in this situation was generally perceived as positive and desirable. In contrast, the additional information in the V2X warnings, in this case, the distance to the broken-down vehicle in meters, was rated less well. Many subjects could not follow the changing additional information or had problems interpreting the information.

The second scenario examined the research question *How do vehicle drivers respond to a false-positive warning when visual feedback is present?* The background of the investigation is the hypothesis that subjects who have already received a V2X warning believe the warning more than the real event and might also lead to react due to the false-positive warning. Regarding accident analysis, such unexpected braking due to the false-positive warning could, in a worst-case scenario, lead to a rear-end collision with a third party driving behind.

However, this hypothesis could not be confirmed. Most subjects executed an appropriate reaction in the form of throttle removal and braking readiness and waited out the situation. In both comparison groups, only 6.20% of the subjects executed a braking action based on the warning. No difference between the groups or dependence on the distance to the vehicle in front was found. The fact that the drivers trust the systems “blindly” despite positive experiences and follow the request by the warning can not be confirmed. Further studies must show whether this will change with a greater user experience for V2X warnings in the future. Nevertheless, the results show that such a warning could trigger an incorrect reaction in rare cases, leading to an accident in the worst case. In the opinion of all the subjects, this scenario could be counteracted by treatment during driving school training and should be included as a scenario. The survey after the study also revealed that more than half of the subjects would turn off the system if there were frequent false alarms. Reliable functionality is, thus, crucial for high-field effectiveness and acceptance of the systems. In general, the design of V2X warnings can be further improved, especially to communicate the change in additional information better. Here, further studies could investigate whether additional sounds, such as different intensities per information change, would bring an advantage.

It is important to note that the results and the findings based on them are purely derived from a simulation with specific scenarios and warning concepts and may differ in reality. In addition, only 32 people were analyzed, none of whom had any experience with V2X prior to the study. These limitations should always be considered when further utilizing the results and findings from this study.

Regarding the accident analysis, it can be concluded that a separate consideration of the type of warning (V2X- or sensor-based) does not have to be distinguished. Also, there are no significant differences concerning the reaction times to be applied, assuming best-case conditions. A possible accident causality, for example, due to an unexpected braking triggered by a false-positive warning of the driver, is also unlikely but possible. To clarify such questions beyond doubt and with legal certainty, it would be advantageous if the occurrence of a V2X warning were also stored in the vehicle’s event data recorder (EDR) [30]. According to the current state of the art, witness-independent proof of the occurrence of a V2X warning is not possible. An extension of the EDR could significantly improve this circumstance and would contribute to an improvement in accident investigation.

## Figures and Tables

**Table 1:** Technical data of the driving simulator [20].

Parameter	Value
Speed (linear)	±0.7 m/s
Speed (rotational)	$\pm 30^{\circ }/$s
Acceleration (linear)	±10 m/s^2^
Acceleration (rotational)	$\pm 150^{\circ }$/s^2^
Cut-off Frequency	5 Hz
Yaw/Pitch/Roll Angle	$\pm 25^{\circ },\pm 20^{\circ },\pm 20^{\circ }$

**Table 2:** Recorded measurement parameters per subject during the study run.

Parameter	Recording
Throttle Position	CarMaker
Brake Pedal Position	CarMaker
Steering Wheel Angle	CarMaker
Steering Wheel Velocity	CarMaker
Steering Wheel Acceleration	CarMaker
Distance Driven	CarMaker
Vehicle Velocity	CarMaker
Vehicle Long. Acceleration	CarMaker
Vehicle Lat. Acceleration	CarMaker
Yaw Angle	CarMaker
Yaw Rate	CarMaker
Yaw Acceleration	CarMaker
Time	CarMaker
Eye Tracking	Cameras
Driving Experience	Recruiting Questionnaire
Simulation Experience	Questionnaire

**Table 3:** Metric for classifying and evaluating the reaction behavior/types of the subjects.

Value	Description
0	No reaction
1	Steering
2	Braking
3	Accelerating
4	Steering and Braking
5	Steering and Accelerating
6	Down from throttle
7	Down from throttle and ready to brake

## Data Availability

The data presented in this study are available on request from the corresponding author. The data are not publicly available due to privacy restrictions.

## Abbreviations

The following abbreviations are used in this manuscript:

ANOVA	Analysis of Variance
CITS	Cooperative Intelligent Transport System
EDR	Event Data Recorder
EEBL	Electronic Brake Lights
EVW	Emergency Vehicle Warning
MQTT	Message Queuing Telemetry Transport
RWW	Roadworks warning
TCW	Traffic Condition Warning
V2I	Vehicle-to-Infrastructure Communication
V2P	Vehicle-to-Person Communication
V2V	Vehicle-to-Vehicle Communication
V2X	Vehicle-to-Everything Communication
